# Psychometric Evaluation of the Arabic Version of the Swedish National Diabetes Registers Instrument for Patient-Reported Experience and Outcome Measures

**DOI:** 10.3390/healthcare14010107

**Published:** 2026-01-01

**Authors:** Nizar Alsubahi, Ahmed Alzahrani, Fahad Alhazmi, Roba Alhaifani, Mohannad Alkhateeb

**Affiliations:** 1 Department of Health Service and Hospital Administration, Faculty of Economics and Administration, King Abdulaziz University, Jeddah 21589, Saudi Arabia; 2 Maastricht University Medical Center, Department of Health Services Research, Faculty of Health, Care and Public Health Research Institute—CAPHRI, Medicine and Life Sciences, Maastricht University, P.O. Box 616, 6200 MD Maastricht, The Netherlands; 3 Department of Health Services Management and Hospitals, College of Business, King Abdulaziz University, Rabigh 21589, Saudi Arabia; 4 Institute of Medicine, School of Public Health and Community Medicine, University of Gothenburg, 40530 Gothenburg, Sweden

**Keywords:** diabetes, Patient-Reported Experience Measures, Patient-Reported Outcome Measures, Swedish national diabetes registers, psychometric validation, Arabic version, Value-Based Healthcare, Vision 2030, Saudi Arabia

## Abstract

**Background:** Healthcare quality is increasingly dependent on the patients’ perspective to ensure care aligns with patients’ needs and experiences, especially for those living with chronic conditions such as diabetes. The Swedish National Diabetes Register instrument (NDR) combines patient-reported experiences and outcomes to evaluate patient-centered diabetes care, but it has not yet been accessible in Arabic. This study aimed to assess the validity and reliability of the Arabic version of the Swedish National Diabetes Register questionnaire among patients with diabetes. **Methods:** A cross-sectional study was carried out from July to August 2023 at 47 primary healthcare centers in Saudi Arabia, involving 594 adult patients with diabetes. Reliability was measured with Cronbach’s alpha and composite reliability. Construct validity was tested using confirmatory factor analysis, and discriminant validity was assessed through the Heterotrait–Monotrait Ratio. Data analysis was performed using SPSS (version 28) and the lavaan package in R (version 4.3.2). **Results**: The Arabic version showed high internal consistency, with Cronbach’s α ranging from 0.716 to 0.886 and CR between 0.663 and 0.855. It also exhibited good model fit indices, including χ^2^/df of 2.72, RMSEA of 0.054, SRMR of 0.073, and a CFI above 0.90. All item loadings were statistically significant (*p* < 0.01). The HTMT values were below 0.85, confirming adequate discriminant validity. **Conclusions**: The Arabic version of the NDR instrument is a valid and reliable tool for measuring patient-reported experiences and outcomes among Arabic-speaking patients with diabetes, which supports its application in diabetic care across the Arab region. Health policymakers in the region are recommended to incorporate this validated Arabic tool into their national diabetes initiatives.

## 1. Introduction

Today, the increasing global rate of chronic diseases presents significant challenges to healthcare systems that were originally designed to handle acute health conditions [1]. Chronic diseases place a substantial economic burden on individuals, families, and society. Managing chronic diseases incurs considerable costs, which can cause financial hardship for patients and their families [2]. Evidence indicates that implementing patient-centered care can effectively guide the management of individuals with chronic diseases, potentially resulting in improved health outcomes [3]. Consequently, healthcare systems are placing a greater emphasis on patients as active participants in their own care, prioritizing outcomes and experiences that are most meaningful to them. This patient-centered approach has established the basis for larger healthcare reforms, including Value-Based Healthcare (VBHC).

Healthcare systems worldwide aim to shift from a volume-focused healthcare model towards VBHC that emphasizes patient value [4,5]. The VBHC model proposal seeks to address healthcare costs concerning their ability to improve patient outcomes [6]. To capture outcomes that genuinely mirror the patient’s view, Patient-Reported Experience Measures (PREMs) and Patient-Reported Outcome Measures (PROMs) are becoming more commonly used to evaluate the quality of chronic disease specialty in diabetic care [7]. PROMs evaluate various outcomes, including physical performance, social functioning, psychological well-being, symptom severity, disability, and impairment from the patient’s perspective. In contrast, PREMs specifically focus on the patient’s experience of care [8]. These measures can be used to support diagnosis, track treatment and patient progress, enhance communication between patients and health care providers, and promote shared decision-making [9].

Diabetes is a long-term condition that impacts millions of people worldwide, projected to reach approximately 643 million by 2030 [10]. The limited scope of health services, combined with the increasing prevalence of diabetes, results in inadequate diabetes care despite many medical centers, clinics, and associations working to address this chronic condition. Recent national statistics from several Arab countries indicate that the prevalence of diabetes is 13.9% in Iraq [11,12], 5.4% of individuals aged 20 to 79 in Yemen [13], 15.4% in Gaza, the West Bank, and East Jerusalem in Palestine [14], 12.6% in Syria [15], and 14.6% among individuals in Lebanon [16]. Moreover, according to data from the World Health Organization (WHO) [13], the Kingdom of Saudi Arabia has the second-highest diabetes prevalence in the Middle East and ranks seventh globally, with national estimates suggesting that about 18–25% of adults are living with diabetes [17].

Given the increasing burden of diabetes across the Gulf region and the strain these factors place on health systems, such as increased service demands and workforce shortages [18,19], there is an urgent need to develop systematic approaches for monitoring and enhancing the quality of diabetes care. National and regional diabetes registries have been demonstrated to be effective tools for assessing clinical outcomes, guiding policy decisions, and improving care delivery [20]. The NDR has established itself as an essential component of quality assurance and enhancement in diabetes care [21]. Implementing PROM and PREM, the register initially developed a disease-specific questionnaire that evaluated patients’ abilities and perceptions of their diabetes care experience, capable of detecting changes over time and differences among patient subgroups [22]. This instrument is an effective measuring tool that was developed for use in diabetic care [3]. The instrument focuses on key factors affecting the quality of diabetes care from the patient’s viewpoint. PREMs include aspects such as (i) support from diabetes care providers, (ii) access to and satisfaction with medical devices and treatments, while PROMs address (i) overall health and emotional well-being, (ii) concerns related to diabetes, (iii) self-management skills, (iv) obstacles to daily activities, and (v) social support.

Although this tool is highly valuable, it has not been translated into any languages other than the official English translation developed by the original authors, and psychometric testing has been conducted using English versions. The Swedish National Diabetes Register questionnaire, which covers both PREMs and PROMs, has proven to be a valid and reliable measure for evaluating the quality of diabetes care and patient outcomes. Although the Swedish National Diabetes Register questionnaire is a valid tool for evaluating diabetes care, no efforts have been made to translate or culturally adapt it into Arabic for use in Arab countries. Therefore, this study aimed to assess the validity and reliability of the Arabic adaptation of the Swedish National Diabetes Register questionnaire, which is designed to capture patient-reported experiences and outcomes in individuals with diabetes.

## 2. Method

### 2.1. Study Design

This observational study used a face-to-face interviewer-administered questionnaire between July and August 2023 at the 47 primary healthcare centers associated with the first and second clusters in Jeddah, Saudi Arabia.

### 2.2. Participants and Sampling Strategy

The study included patients aged 18 and older, as they could give informed consent and clearly express their healthcare experiences, which helps ensure data accuracy. Participants with diabetes (type 1 and type 2) who were clinically stable, had complications such as uncontrolled HbA1c levels, and were able to visit Primary Health Centers (PHCs) during the study period were also included. However, people with cognitive disabilities, communication challenges, and language barriers were excluded.

Simple random sampling was conducted within each center. At the reception, research assistants approached all patients diagnosed with diabetes and invited them to participate. Over 800 patients were initially invited across all PHCs. Each day, a random list was generated from the appointment registry at each center, and 10–15 eligible patients were selected and invited to complete the interview.

### 2.3. Data Collection

Data were collected through the main author and research assistants. All team members were certified health professionals experienced in conducting structured interviews and surveys. Before fieldwork, the authors conducted comprehensive training and reviewed the research protocol to ensure standardized procedures. Data collection was conducted through face-to-face interviews in private rooms across PHCs. Daily debriefing meetings were held throughout the data collection period to monitor progress, address challenges, and maintain data quality. Conducting interviews in private settings enhanced confidentiality and accuracy, while standardized interviewer training minimized bias and ensured consistency across all sites.

### 2.4. Study Instrument

This research employed the Swedish National Diabetes Register Questionnaire as its instrument [21]. PREMs evaluate two main aspects related to patient experience. The first aspect concerns support from diabetes care providers (9 items). This assesses the support patients receive, including how easy it is to contact providers, the ability to see a nurse or physician as often as needed, the convenience of appointment times, continuity of care with the same providers, and the opportunity to discuss important issues during appointments. The second aspect concerns satisfaction with medical devices and treatment (3 items). This assesses satisfaction with blood sugar monitoring devices, insulin delivery tools, and overall contentment with medication and treatment. All items were evaluated using a 5-point Likert scale: 1 = Not applicable; 2 = No, never; 3 = No, not often; 4 = Yes, usually; 5 = Yes, always. A higher score reflects a better healthcare experience. PROMs evaluate five aspects of patient health outcomes. The first aspect concerns feelings over the past four weeks (5 items), including general mood, sleep quality, depression, diabetes-related distress, and self-control over diabetes, rated on a 5-point scale where higher scores mean better outcomes. The second aspect pertains to worries (3 items), assessing patient concerns about low blood sugar, high blood sugar, and diabetes complications, with higher scores reflecting fewer worries. The third dimension relates to the capabilities to manage diabetes (5 items). This evaluates patients’ confidence in managing their condition, which includes their knowledge, routine care, coping with disruptions, healthy eating, and staying active. Higher scores indicate greater confidence in managing diabetes capability. The fourth aspect addresses the obstacles to living as preferred (5 items). This explores how diabetes, blood sugar changes, and their effects on relationships create barriers to patients’ ideal lifestyles, with higher scores reflecting fewer obstacles. Finally, the fifth aspect concerns support from others (3 items). This assesses the help patients get from family, friends, their daily contacts, and fellow diabetes patients. Higher scores reflect greater support.

### 2.5. Translating the Instrument

**Figure 1:**
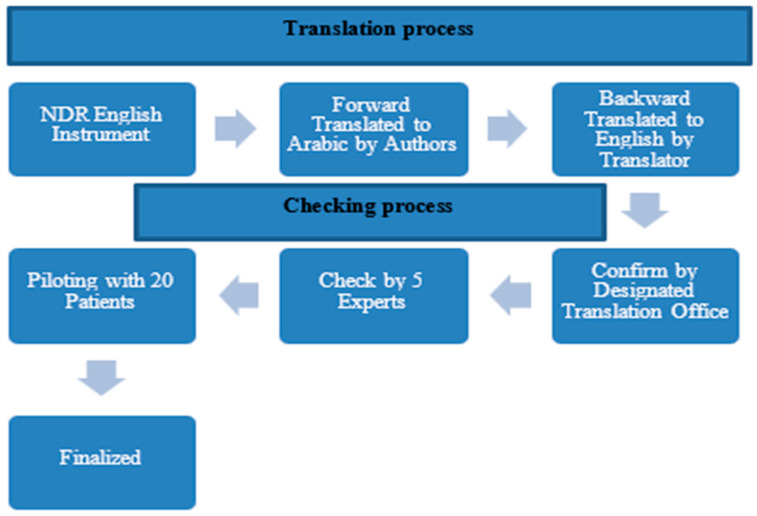
Translation process.

The instrument translation and cultural adaptation were carried out through a structured multistep process (see Figure 1). The original NDR instrument was created in Swedish, but the Swedish source is not publicly available. The developers provide the validated English version as the official reference for cross-cultural adaptation and international use, so the Arabic translation in this study was prepared from the English version. The Arabic adaptation involved a forward–backward translation method, followed by review by an expert panel, adhering to established cross-cultural adaptation guidelines. A forward translation of the English NDR questionnaire into Arabic by two authors. A backward translation into English was then performed by two certified translators who were blinded to the original version. The international translation office then confirmed the instrument. An expert panel, including professors from King Abdul-Aziz University specializing in public health and family medicine consultants from the Ministry of Health, reviewed the questionnaire to confirm its content validity. They also evaluated the semantic equivalence by examining both versions. The revised Arabic version was additionally reviewed and verified by a designated translation office. Ultimately, the pre-final version was tested with 20 patients to assess clarity, understanding, and cultural suitability before completing the final version. This ensured the translated questionnaire’s fidelity and preserved the instrument’s validity throughout the study. The survey was conducted in Arabic, and Qualtrics software (https://www.qualtrics.com/) was used to create questionnaires for collecting data from participants.

### 2.6. Data Analysis

Data analysis was conducted with SPSS version 28 and the lavaan package in R (version 4.3.2). The study performed a Confirmatory Factor Analysis (CFA) to assess the psychometric properties of the measurement scales and examine the relationships between variables. Responses labeled as ‘Not applicable’ were considered a form of structural missingness, meaning the item was irrelevant to the respondent rather than a typical missing value (MCAR, MAR, or MNAR). Consequently, N/A responses were omitted from psychometric analyses. Convergent validity was assessed by analyzing composite reliability (CR) and standardized factor loadings [23]. Convergent validity criteria are satisfied if CR exceeds 0.734 or if the standardized factor loadings are significant [24]. Also, the Average Variance Extracted (AVE) value ranged from 0.318 to 0.657, while the internal consistency reliability was evaluated with Cronbach’s alpha, with values exceeding 0.7 indicating satisfactory reliability [24]. The Comparative Fit Index (CFI) indicated an adequate model fit based on the standard threshold of 0.90 [25]. The thresholds were applied to assess the adequacy of the measurement model. Regarding discriminant validity, we employed the Heterotrait–Monotrait Ratio (HTMT) criterion [26] in which HTMT ratios are acceptable, below 0.85.

## 3. Results

### 3.1. Participation

Over 809 invited patients, 594 completed the questionnaire, yielding a response rate of 73.4%. The remaining 215 patients (26.6%) did not participate due to time constraints and scheduling conflicts. The sociodemographic characteristics showed that 49.3% of respondents were male and 50.7% female. Most respondents (254) fell within the 41 to 60 age range, with 42.8% having completed secondary education (43.3%). The majority were married (68%), unemployed (45.3%), and had a low monthly income (60.9%). Additionally, 85.4% of participants were Saudi citizens, 72.6% did not smoke, 71.5% had type 2 diabetes, and 44.4% had been diagnosed with diabetes within the past 15 years.

### 3.2. Contract Validity

**Figure 2:**
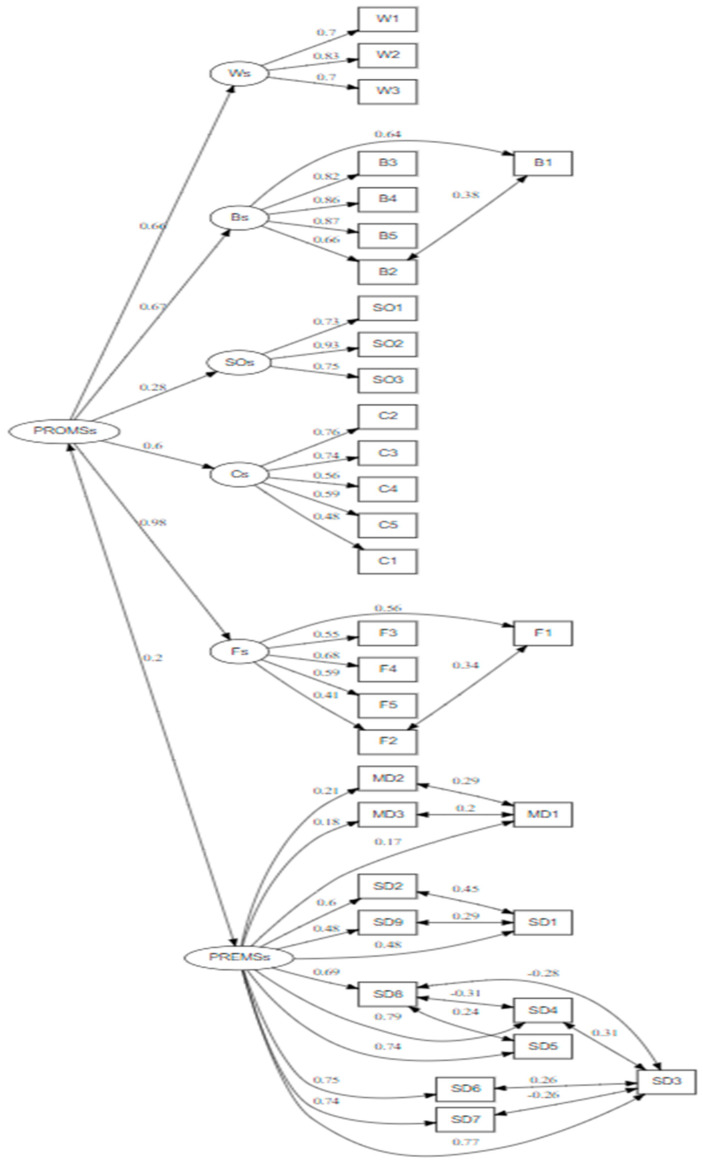
Confirmatory Factor Analysis of the Arabic instrument.

Construct validity was assessed using CFA, with a Root Mean Square Error of Approximation (RMSEA) of 0.054 and a Standardized Root Mean Square Residual (SRMR) of 0.073, both meeting recommended thresholds. Incremental fit indices confirmed adequate model fit, with a Comparative Fit Index (CFI) of 0.904 and a Tucker–Lewis Index (TLI) of 0.892. The Goodness-of-Fit Index (GFI) was 0.883, which is acceptable considering the model’s complexity. Although the chi-square test was significant (χ^2^ = 1273.911, df = 468), the χ^2^/df ratio of 2.72 also suggests a good fit, as ratios below 3 are generally acceptable. Figure 2 displays the CFA model, showing the links between the latent constructs (PREMs and PROMs) and their observed indicators. All standardized factor loadings were statistically significant (*p* < 0.01), ranging from 0.172 to 0.913, with lower values observed in the medical device satisfaction subscale. Convergent validity was evaluated using the AVE as shown in Table 1. AVE values varied from 0.318 to 0.657, indicating acceptable convergence for several constructs, while some showed lower values due to limited variance and ceiling effects in certain items. Modification indices were reviewed to identify possible improvements in model fit; however, no modifications were found. Therefore, no model specification was carried out, and the original factor structure was preserved. Despite this variation, all loadings were significant, indicating acceptable reliability and convergent validity. Each path coefficient indicates the standardized factor loading, reflecting the strength and direction of the association between variables. The model overall aligns with the validation of the construct validity of the Arabic version. Based on the original NDR instrument and SEM terms, each item follows the standard measurement equation: x = Λξ + δ, where Λ represents the factor loadings and δ indicates measurement errors.

### 3.3. Reliability Analysis

Table 2 shows that Cronbach’s alphas, which are used to test the reliability and consistency of the instrument [27]. The PROMs subscales demonstrated reliability ranging from acceptable to excellent, with Cronbach’s alphas from 0.758 to 0.888. The Support from Providers subscale of PREMs showed high reliability (α = 0.886), whereas the Medical Treatment subscale had lower internal consistency (α = 0.398). However, the total PROMs and PREMs scales displayed good reliability, with alpha values of 0.729 and 0.844, respectively. As shown in Table 1, Composite Reliability (CR) values further confirmed the reliability of the constructions, with PROMs domains ranging from 0.663 to 0.855 and the PREMs scale showing a CR of 0.819, corresponding to the acceptable level as recommended by Hair, Sarstedt [28]. All items had statistically significant factor loadings, ranging from 0.172 to 0.913, indicating that each item contributed meaningfully to its construct, despite some variation in loadings.

### 3.4. Discriminant Validity

Construct-level discriminant validity was evaluated through the Heterotrait–Monotrait (HTMT) ratio. Table 3 presents the HTMT values used to assess discriminant validity. These values demonstrate acceptable discriminant validity, as all are below the widely recognized cutoff of 0.85. The findings confirm that the constructs are sufficiently distinct yet still capture important relationships.

## 4. Discussion

### 4.1. Statement of Principle Findings

This study aimed to evaluate the psychometric properties of the Arabic version of the NDR instrument, which is designed to assess PREMs and PROMs in patients with diabetes in Saudi Arabia. The results show that the Arabic version of the instrument has good reliability, convergent validity, and discriminant validity, confirming it as a dependable and valid tool for Arabic-speaking countries. The NDR instrument was translated following guidelines from the World Health Organization for cultural adaptation and translation of original tools [11], and a culturally equivalent instrument was created, though employing this method during the translation of an existing tool [29].

The Confirmatory Factor Analysis indicated an acceptable model fit, as evidenced by fit indices like RMSEA, SRMR, and CFI meeting the recommended standards thresholds. Cronbach’s alpha and composite reliability scores for both PREMs and PROMs surpassed the acceptable thresholds, confirming internal consistency. The lower factor loadings in the “satisfaction with medical device” subscale related to PREMs appear to result from ceiling effects, as participants consistently provided high ratings. Nevertheless, the reliability indices stayed acceptable, and the items were kept to ensure content validity and maintain consistency with the original NDR instrument. This pattern aligns with the initial validation of the NDR questionnaire, which demonstrated acceptable reliability and construct validity in both PROM and PREM domains [21,30]. Similar ceiling-related patterns were observed in the original NDR psychometric assessment, where several items showed decreased agreement, which was due to skewed distributions rather than ineffective item performance. It is advisable to retain these items when the content validity is high, and maintaining comparability with the source instrument is essential.

These findings are consistent with those from the English versions of the instrument, which also showed strong psychometric properties [21]. This indicates that the NDR instrument’s structure and conceptual framework can be used across different cultural contexts, provided proper translation and adaptation processes are employed. These findings align with earlier research that highlights the importance of employing validated patient-reported measures to evaluate diabetes care quality and to ensure that patients’ perspectives are incorporated into healthcare improvement initiatives [21,22].

### 4.2. Implication for Policy, Practice, and Research

Patients with diabetes need ongoing, holistic medical care along with diverse management strategies [3]. PREMs and PROMs offer complementary insights into patient experiences and outcomes, both of which are vital components of the overall assessment of VBHC [7]. PREMs in this study focused on the support from diabetes care providers and satisfaction with medical devices and treatments, highlighting important factors for patient engagement and satisfaction. PROMs, meanwhile, measured emotional well-being, concerns, self-management skills, daily life challenges, and social support—factors impacting long-term disease management and quality of life. These results highlight the importance for healthcare systems in the Arab region to implement comprehensive evaluation frameworks that encompass both patient experience and outcome measures to improve diabetes care [7,20].

The successful validation of this instrument in Arabic is especially significant due to the high rates of diabetes in the Middle East [31]. Integrating this tool into national diabetes and electronic health records can improve the monitoring of patient outcomes, support benchmarking among healthcare providers, and inform policy decisions to enhance chronic disease management. Additionally, it provides researchers and practitioners with a standardized way to evaluate patient perspectives, which are frequently underrepresented in clinical assessments. The Arabic tool demonstrated robust psychometric properties. Therefore, it can be used in the Arab region. Also, it can be used to improve the quality of care in chronic disease.

### 4.3. Strengths, Limitations, and Future Research

To the best of our knowledge, this is the first study of its kind to have been conducted in the Arab region. A key strength of this research is the collection of data from 47 primary healthcare centers in Saudi Arabia, representing a diverse patient population in age, gender, education, and socioeconomic status. This wide sampling improves the representativeness of the results and helps ensure that the Arabic version of the instrument is applicable across various primary care environments. However, this study also has some limitations. Although the data were gathered from several centers, all were within Jeddah, Saudi Arabia, which may limit the generalizability of the results to other Arab populations or to varying levels of care. Future research should replicate these findings across Arabic-speaking regions and in different levels of care, including secondary and tertiary settings. Ceiling effects seen in some PREMs items may have limited response variation and led to lower factor loadings, but overall reliability and content validity stayed acceptable. Using self-reported measures can lead to recall and response biases. Future research could use longitudinal designs to enhance cross-country relevance and evaluate the instruments’ consistency and effectiveness over time.

## 5. Conclusions

The Arabic adaptation of the Swedish National Diabetes Register instrument shows robust psychometric qualities, confirming its suitability and reliability for assessing patient-reported experiences and outcomes in Arabic-speaking individuals with diabetes. Considering the rising rates of diabetes in the Arab region, this validated Arabic tool is a significant step toward enhancing patient-centered care and reinforcing the principles of VBHC. Integrating PREMs and PROMs into national diabetes initiatives and healthcare organizations is crucial for better monitoring of patient experiences and outcomes, improving care quality, and promoting transparency in health system performance. Health policymakers in Saudi Arabia and neighboring Arab nations should consider integrating this validated Arabic tool into their regular quality monitoring and performance assessment systems. This integration can support the 2030 Saudi Vision, which aims to enhance the overall quality of care across all healthcare facilities. Using standardized patient-reported measures can facilitate benchmarking between healthcare facilities, inform specific interventions, and help meet the objectives of national and regional diabetes strategies focused on enhancing chronic disease management and overall population health.

## Figures and Tables

**Table 1:** Psychometric properties of PREMs and PROMs scales.

Variable	CR	Mean (SD)	AVE	Item	Mean (SD)	Factor Loadings *
PREMs	0.819	4.13 (1.28)	0.357	SD1	4.35 (0.97)	0.490
				SD2	4.04 (1.2)	0.608
				SD3	3.53 (1.44)	0.754
				SD4	3.72 (1.37)	0.782
				SD5	3.47 (1.44)	0.741
				SD6	3.81 (1.28)	0.756
				SD7	4.00 (1.13)	0.742
				SD8	3.8 (1.32)	0.688
				SD9	4.21 (1.05)	0.480
				MD1	4.13 (1.28)	0.172
				MD2	3.28 (1.76)	0.221
				MD3	4.38 (1.01)	0.195
PROMs feels	0.663	3.52 (0.92)	0.318	F1	4.13 (0.84)	0.555
				F2	3.92 (0.95)	0.403
				F3	2.72 (1.05)	0.551
				F4	2.59 (1.01)	0.684
				F5	4.24 (0.75)	0.590
PROMs Worries	0.786	2.28 (1.09)	0.552	W1	2.47 (1.1)	0.714
				W2	2.24 (1.08)	0.815
				W3	2.13 (1.09)	0.695
PROMs Capabilities	0.758	4.25 (0.788)	0.375	C1	4.18 (0.83)	0.772
				C2	4.37 (0.72)	0.407
				C3	4.2 (0.75)	0.457
				C4	4.31 (0.76)	0.682
				C5	4.19 (0.88)	0.659
PROMs Barriers	0.855	2.77 (0.992)	0.602	B1	2.57 (0.99)	0.632
				B2	2.94 (1.04)	0.657
				B3	2.91 (0.97)	0.816
				B4	2.77 (0.97)	0.862
				B5	2.68 (0.99)	0.878
PROMs Support	0.853	4.33 (0.867)	0.657	SO1	4.38 (0.81)	0.738
				SO2	4.28 (0.86)	0.913
				SO3	4.35 (0.84)	0.769

* All factor loadings were significant at *p*-value < 0.01.

**Table 2:** Reliability analysis for the instrument.

Patient-reported outcome measures PROMs		
Variable	Number of items	Cronbach’s alpha
Feeling	5	0.843
Worrying	3	0.785
Capabilities	5	0.758
Barriers	5	0.888
Support from others	3	0.845
Total PROMs	21	0.729
Patient-reported experience measures PREMs		
Variable	Number of items	Cronbach’s alpha
Support from the provider	9	0.886
Medical treatment	3	0.398
Total PREMs	12	0.844

**Table 3:** Heterotrait–Monotrait (HTMT) Construct-Level Discriminant validity.

Construct	1	2	3	4	5	6
Patient-reported outcome variables						
W	1					
B	0.617	1				
SO	0.072	0.064	1			
C	0.321	0.316	0.389	1		
F	0.564	0.599	0.234	0.572	1	
Patient-reported experience	0.133	0.17	0.521	0.346	0.234	1

## Data Availability

The original contributions presented in this study are included in the article. Further inquiries can be directed to the corresponding author.

## Abbreviations

PREMs	Patient-Reported Experience Measures
SD	Support from Providers
MD	Medical Devices
PROMs	Patient-Reported Outcome Measures
W	Worring
F	Feeling
B	Barriers
C	Capabilities
SO	Support from Others
VBHC	Value-Based Healthcare
WHO	World Health Organization
CFA	Confirmatory Factor Analysis
CR	Composite Reliability
CFI	Comparative Fit Index
RMSEA	Root Mean Square Error of Approximation
